# Evaluation of dietary intake of lactating women in China and its potential impact on the health of mothers and infants

**DOI:** 10.1186/1472-6874-12-18

**Published:** 2012-07-16

**Authors:** Haijiao Chen, Ping Wang, Yaofeng Han, Jing Ma, Frederic A Troy, Bing Wang

**Affiliations:** 1 School of Medicine, Xiamen University, Xiamen, 300136, China; 2 Department of Biochemistry and Molecular Medicine, University of California School of Medicine, Davis, CA, 95616, USA; 3 School of Molecular Bioscience, The University of Sydney, Sydney, NSW 2006, Australia

**Keywords:** Nutrient intake, Lactating women, Chinese RNI and China

## Abstract

**Background:**

Optimal nutrition for lactating mothers is importance for mother and infants’ health and well-being. We determined the nutrient intake and dietary changes during the first 3-month of lactation, and its potential effect on health and disease risk.

**Method:**

Personal interviews were conducted to collect a 24h diet recall questionnaire from 199 healthy lactating women in the postpartum days 2, 7, 30, 90 and healthy 58 non-pregnant women served as the controls.

**Results:**

We found in lactating women (1) the mean daily energy and carbohydrate intake was lower than that of the Chinese Recommended Nutrient Intake (RNI, 2600 Kcal, 357.5 ~ 422.5g) by 11% ~ 17% and 33% ~ 49%, respectively; (2) the fat intake increased from 3% to 13%, which was 9 ~ 77% higher than the RNI (57 ~ 86.7g); (3) the protein intake exceeded the RNI of 85g by 32 ~ 53%; (4) the total calories consumed from carbohydrate (39%-44%), fat (34% ~ 42%) and protein (20%-23%) failed to meet Chinese RNI (5) the intake of vitamin C, B1, folate, zinc, dietary fiber, and calcium was 5% ~ 73% lower than the RNI while vitamin B_2_, B_3_, E, iron and selenium intake was 20% to 3 times higher than the RNI. Nutrient intake in the control group was lower for all nutrients than the recommended RNI.

**Conclusion:**

Lactating women on a self-selected diet did not meet the Chinese RNI for many important micronutrients, which may influence the nutritional composition of breast milk and thus impact the potential health of mothers and infants. RNI should consider the regional dietary habits and culture. A single national RNI is not applicable for all of China. Nutritional education into the community is needed.

## Background

The nutrient intake of lactating women is one of most important determinants of woman’s health, well-being and the ability for long-term successful breastfeeding. Human lactation is a natural process, which is well established to provide many healthy benefits for both mothers and their infants. Lactation also has many favourable effects on women, including reducing the incidence of type 2 diabetes, metabolic syndrome [1,2], cardiovascular disease [3,4] and cancer [5]. The nutrient intake of lactating women affects the nutrient content of breast-milk and maternal health [6]. As noted, many essential nutrients are secreted into breast milk and represent a significant proportion of nutrient intake in the maternal diet, including docosahexaenoic acid (DHA) [7-9], most vitamins including vitamin B2 [10], vitamin A [11] and vitamin D [12,13]. Thus, nutritional requirements for lactating women are higher compared to women who do not breastfeed [14,15].

Perinatal health of the infants are closely linked to the well-being of mothers [16,17]. The nutrients that infants receive from breast milk, and the mother's overall physical and mental health during breastfeeding, are all factors that affect the baby’s early heath and will continue to influence their health into later life [18]. The nutritional content of human breast-milk has been shown to enhance many biochemical and metabolic pathways critical to the development of most organ systems including the digestive, cardiovascular, pulmonary, immune, endocrine and nervous system [19-22]. These metabolic events are vital for maintaining the normal function and health of newborns, particularly those born prematurely or with a low birth weight, and that often persist long after nursing [19,23].

Less definitive information is available on what lactating mothers currently eat and its impact on maternal nutrition and wellness in China. In particular the current prevalence of chronic diseases including obesity, cardiovascular disease, hypertension, diabetes mellitus and a number of other diseases have increased overall in China over pass 30 years rapid economic development [24,25]. Lactating women’s nutrient intake is influenced by Westernized lifestyle that is shifting disease patterns at an alarming rate in China towards a profile exceeding that is seen in the more developed countries. It is estimated, for example, that eight of ten deaths in China now result from a chronic disease. In the U.S. this figure is seven out of ten deaths (WHO). Surprising, however, there is little information that systematically evaluates the nutrient intake of lactating women in China during the first three months of lactation. The specific aim of this study was to systematically evaluate the nutrient intake and dietary changes of lactating women in China during the first three months of lactation. We also compared the nutrient intake of lactating women to a control group of non-pregnant and non- lactating woman. We address the major regional and cultural differences in China that makes it difficult to prescribe a “one RNI fits all” recommendation for a country of this size with so many disparate ethnic and cultural groups.

## Methods

Participants were recruited through flyers advertising the study at the Obstetrician’s office in the Women’s and Children’s Hospital, the largest teaching hospitals for healthy women and lactating mothers in Fujian province. One hundred ninety nine healthy and exclusively breastfeeding women aged 23 to 38 years old were initially enrolled in the study beginning on postpartum day two. Of these 199, 195, 196, 173 of the lactating women completed 24h food intake recording at 4 time points, representing on days 2, 7, 30 and 90 of lactation, respectively. These four time points are referred to in the text as the “3 month test period”, which represents the first 90 days of lactation. A trained and experienced researcher interviewed each participant in their home and recorded the amount and type of food consumed by weighing on a portable digital scale. Participants were provided with a listing of more than 140 food items to be marked in each category on the questionnaire, and to indicate the frequency of daily consumption. The questionnaires served as the basis for determining the intake of basic nutritional components, vitamins, minerals and other dietary supplements. The questionnaires, data collection and quality control procedures are based on China Health and Nutrition Survey guideline and method http://www.cpc.unc.edu/projects/china.

One hundred seventy three of the lactating women completed the 24h dietary intake questionnaire for all four-time periods during the 3 months study. Major reasons for women discontinuing the food recording included “they were too busy or tired, time constraints and other family responsibilities”. Fifty-eight healthy women were recruited as the control group and collected data once on the 90th day of data collection for the lactating women. The selection criteria for the healthy women were non-pregnant, non- lactating and not on any medications. The control and lactating women were instructed not to change their dietary or physical activity during the study. The 24h diet intake questionnaire for the control subjects was collected and analyzed in the identical way as the test group of lactating mothers. All participants’ information and food intake was registered in a computer database. The study protocol was approved by the Human Ethics Committee of Xiamen University. All invited participants provided written informed consent before inclusion in the study.

The food intake were analysed by trained researchers using the China Food Composition to calculate individual nutrient intakes of the 1506 different foods and 31 nutrients [26]. Energy and selected nutrient intakes were analyzed using Software for “Intake Distribution Estimation” based on Chinese food nutrition composition [14,15]. The general characteristics of subjects during the 3 months test period that were used to calculate individual nutrient intakes are summarised in Table 1.

**Table 1:** **General characteristics of study participants**^**1**^

	**Lactating women**				**Controls**
	**2nd day (n = 199)**	**7th day (n = 195)**	**30th day (n = 196)**	**90th day (n = 173)**	**(n = 58)**
Age (yrs)					
– 20-25	47	44	36	39	4
– 26-30	125	124	129	112	36
– 31-35	26	26	30	21	16
– >35	1	1	1	1	2
Height (cm)	160.2 ± 4.2	160.4 ± 4.1	160.1 ± 4.3	159.9 ± 4.0	159.0 ± 3.5
Weight (kg)	60.1 ± 6.4^b^	59.1 ± 6.6^b^	58.6 ± 6.7^b^	58.9 ± 5.5^b^	52.9 ± 3.8^a^
BMI (kg/M^2^)	23.4 ± 2.0^b^	22.9 ± 2.2^b^	22.8 ± 2.1^b^	23.0 ± 1.9^b^	20.9 ± 1.1^a^
First child	Yes	Yes	Yes	Yes	N/A
Residence					
– Local	186(93.5)	185(94.9)	187(95.4)	168(97.1)	55(94.8)
– Immigrant	13(6.5)	10(5.1)	9(4.6)	5(4.8)	3(5.2)
Ethnic group					
– Han	195(98.2)	189(97.1)	186(95.0)	173(100)	56(96.6)
– Others	4(1.8)	6(2.9)	10(5.0)	0	2(3.4)
Education					
– Middle school	25(12.5)	15(7.5)	20(10.0)	23(13.3)	7(11.9)
– High school	49(24.6)	47(23.9)	34(17.5)	69(40.0)	14(24.3)
– College	60(30.2)	78(40.2)	78(40.0)	46(26.7)	16(27.6)
– University	65 (32.7)	55(28.4)	64(32.5)	35(20.0)	21(36.2)
Smoking	never	never	never	never	never
Drinking	never	never	never	never	occasionally
Regular exercise	no	no	yes	yes	yes
Medication	no	no	no	no	no

^1^Values (mean ± SD) in the same row with different superscript letters (a,b) denotes a significant different, *P <* 0.01. Bonferroni adjustment for multiple comparisons of the lactating women compared with the control group of women and a repeated measures analysis of Greenhouse-Geisser adjustment for asphericity for 4 different time points comparison of the lactating women.

Data were analyzed statistically by two-factor repeated measures ANOVA model for the four time periods, using the Greenhouse-Geisser adjustment for asphericity for the lactating women. Comparisons between lactating women at the four time periods and the normal control group of women were made using a multivariate general linear model for the components of selected nutrients (protein, fat, vitamins etc.), with adjustment for BMI as a covariate. All analyses were completed by using SPSS for Macintosh 19. (SPSS Inc., Chicago). The significance level was set at P < 0.05, and for multiple comparisons, P < 0.01.

## Results

### Subjects

There was no significant difference in age, ethic group, education, smoking, alcohol consumption and family social status between lactating and control women in the study. The mean body mass index (BMI) of the lactating mothers were within the normal range of 22.8 ~ 23.4 (±1.9 ~ 2.2) and did not change significantly during the first 3 months of lactation. The mean BMI (20.9 ± 1.1) for the control group of women was also in the normal range for the recommended Chinese BMI [27,28] and was 9% ~ 10% lower than that of lactating women. The difference in BMI between lactating and control women was highly significant (P < 0.001), as shown in Table 1.

### Consumption of food groups

The mean intake of food groups (g/day) in the lactating and control women during the first 3 months lactation is shown in Table 2. The rank orderings of mean intake for the food groups from high to low in lactating women were animal food, cereals, vegetables, fruits, dairy products, legumes/legumes products, nuts/seeds, fungi and algae. The mean intake of total calories in animal food, flour and flour products in lactating women, showed a significant decrease over time (*P <* 0.005), when the full data set of 199 women was analyzed or in the 173 women who completed the 4 time-points of the study when analysed separately (Table 2 P < 0.01 ~ 0.001). On the 90^th^ day of lactation, ~74% ~ 80% of the total dietary calories, 85% ~ 96% of the animal food, and 20% ~ 42% of the flour and flour products remained at the level found during the first month of lactation. However, the mean intake of vegetables, nuts/seeds, fungi, algae, rice/rice products, dairy products, legumes/legumes products and root and tubers showed a significant increase over time, for the 173 lactating women who had completed the study (Table 2, P < 0.005 ~ 0.0001). Also the intake of most food categories in the control group of women was lower than in the lactating women at all four time periods, either before or after the BMI was adjusted as a covariant (P < 0.005 ~ 0.0001, Table 2), exceptions were vegetables, fruits and legumes and legumes products. However, the mean intake value for animal food in the control group of women was ~35% to 45% lower than the lactating women throughout the 3 mo studies (*P <* 0.0001 Table 2).

**Table 2:** **The mean intake of food groups (g/day) during the first 90 days lactation**^**1**^

	**Lactating women**				**Controls**	
**Food categories**	**2nd day** ** (n = 199)**	**7th day (n = 195)**	**30th day** ** (n = 196)**	**90th day** ** (n = 173)**	**(n = 58)**	**Common varieties**
Pure Calories	57 ± 4^b^	59 ± 3^b^	54 ± 3^ab^	44 ± 2^ac^	33 ± 1^c^	Oil, brown sugar, chocolate
Animal Food	605 ± 25^ab^	659 ± 20^ab^	683 ± 23^b^	580 ± 15^a^	378 ± 22^c^	Pork, beef, fish, Chicken, clam, duck, harslet
Nuts/seeds	8 ± 2^b^	13 ± 3^ab^	12 ± 2^ab^	19 ± 2^a^	1 ± 1^b^	Peanuts, kernels, sesame
Vegetables	99 ± 8^b^	123 ± 7^b^	183 ± 8^a^	218 ± 7^a^	202 ± 11^a^	Lettuce, cabbage, Bok choy, tomatoes, kidney bean
Fungi and algae	7 ± 2^a^	7 ± 1^a^	10 ± 2^a^	18 ± 2^b^	7 ± 3^a^	agrocybe aegerita, lentinus edodes
Fruits	138 ± 17	118 ± 11	164 ± 12	158 ± 8	127 ± 12	Apple, banana, lichee, longan, red date
Cereals	335 ± 16^bc^	355 ± 12^b^	401 ± 10^a^	409 ± 8^a^	293 ± 13^c^	
– Rice and rice products	270 ± 16^a^	287 ± 11^a^	335 ± 11^b^	364 ± 8^b^	261 ± 16^a^	Rice, rice noodles
– Flour and flour products	126 ± 13^b^	89 ± 9^c^	59 ± 6^cd^	25 ± 4^a^	24 ± 5^ad^	Steamed bread, noodles
– Other cereals	15 ± 4^ab^	16 ± 4^ab^	7 ± 2^ab^	19 ± 3^b^	0.4 ± 0.4^a^	Millet, glutinous rice, coix seed
Legumes/legumes products	18 ± 3^b^	21 ± 4^ab^	31 ± 6^ab^	39 ± 7^a^	50 ± 12^a^	Soybean, soybean milk
Dairy products	29 ± 6^b^	42 ± 7^b^	95 ± 12^a^	119 ± 10^a^	39 ± 12^b^	Milk, powdered milk
Root and tubers	0.4 ± 0.3^a^	0.4 ± 0.3^a^	4 ± 2^a^	16 ± 3^b^	0 ± 0^a^	Potato, carrots

^1^Values (mean ± SE) in the same row with different superscript letters (a, b, c, d) are significantly different *P <* 0.05 ~ 0.0001, Bonferroni adjustment for multiple comparisons of the lactating women compared with the control group of women and a repeated measures analysis of Greenhouse-Geisser adjustment for asphericity for 4 different time points comparison of the lactating women.

The rank of animal food intake in lactating women from high to low was meat and meat products (29% ~ 35%), fish/shellfish and seaweeds products (19% ~ 23%), poultry (6% ~ 11%), eggs (7% ~ 9%), and milk/milk products (1% ~ 3%) (Table 3). The intake of meat and meat products and fish/shellfish decreased during the course of lactation, while the consumption of milk and milk products increased. The control group of women had a similar pattern of animal food intake as the lactating women, except that egg intake was higher than poultry and poultry products (Table 3). In lactating women, the total animal food intake contributed about 70% of the total protein consumption throughout the first 3 months of lactation (Table 3). Similar to the lactating women, 73% of the protein intake of the control women came from animal food. Significantly, more than 20% of the protein was from fish/shellfish and seaweed products in both lactating and control group of woman. The overall mean intake of different protein sources was lower in the control women compared to the lactating women either before or after the BMI was adjusted as a covariant (Table 3, *P <* 0.01 ~ 0.0001).

**Table 3:** **Mean animal food intake (g/day) and the percentage of protein intake from different protein sources in lactating and control group of women**^**1**^

	**Lactating women**								**Controls**	
	**2nd day (n = 199)**		**7th day (n = 195)**		**30th day (n = 196)**		**90th day (n = 173)**		**(n = 58)**	
	**Intake**	**% protein**	I**ntake**	**% protein**	**Intake**	**% protein**	**Intake**	**% protein**	**Intake**	**% protein**
Meat and meat products	242 ± 17^b^	30.7 ± 1.5^ab^	227 ± 14^ab^	29.1 ± 1.4^ab^	225 ± 11^ab^	29.9 ± 1.3^b^	226 ± 10^ab^	34.6 ± 1.3^ac^	175 ± 12^a^	41.2 ± 2.2^c^
Poultry and poultry products	89 ± 13^ab^	6.7 ± 1.1^ab^	130 ± 14^b^	10.6 ± 1.2^bc^	110 ± 12^b^	10.1 ± 1.0^c^	53 ± 7^a^	6.0 ± 0.8^ab^	24 ± 6^a^	4.0 ± 1.1^a^
Milk and milk products	29 ± 6^b^	1.0 ± 0.2^a^	42 ± 7^b^	1.1 ± 0.2^a^	95 ± 12^a^	2.2 ± 0.3^c^	119 ± 10^a^	3.2 ± 0.3^d^	39 ± 12^b^	0.9 ± 0.4^ac^
Eggs	69 ± 6^ab^	7.5 ± 0.7^ab^	88 ± 6^b^	9.4 ± 0.7^b^	79 ± 4^b^	8.3 ± 0.5^ab^	59 ± 3^a^	6.8 ± 0.4^ac^	29 ± 4^c^	4.6 ± 0.8^c^
Fish/shellfish Seaweeds products	176 ± 15^b^	23.0 ± 1.7	172 ± 12^ab^	21.4 ± 1.4	176 ± 11^b^	21.5 ± 1.3	124 ± 9^a^	19.1 ± 1.3	118 ± 9^ab^	22.7 ± 1.7

^1^Values (mean ± SE) in the same row with different superscript letters (a, b, c) are significantly different (P < 0.05 ~ 0.0001). Bonferroni adjustment for multiple comparisons of the lactating women compared with the control group of women (P < 0.01) and a repeated measures analysis of Greenhouse-Geisser adjustment for asphericity for 4 different time points comparison for the lactating women.

### Energy and selected nutrient intake

The mean energy and selected nutrient intake of lactating women and the control group of women is shown in Table 4. In the 173 lactating women, the mean energy intake did not change significantly over time (Table 4, P > 0.05), but the level of energy intake was 11% ~ 17% lower than that recommended by the Chinese RNI (2,600 kcal/day) at the four different time points. The control group of women had a 38% lower mean energy intake than that recommended by the RNI for a moderate level of physical activity (2,300 Kcal/day). Lactating women had about a 35% higher energy intake than the control group throughout the 3 month study (*P* < 0.05 ~ 0.001), whether justified for BMI as a covariant or not.

**Table 4:** **Mean energy (Kcal) and selected nutrients intake in lactating women during the first 90 days of lactation and the controls**^**1**^

	**Lactating women**					**Controls**	
	**RNI for lactating Women**	**2**^**nd**^** day (n = 199)**	**7**^**th**^** day (n = 195)**	**30**^**th**^** day (n = 196)**	**90**^**th**^** day (n = 173)**	^**2**^**RNI for normal women**	**(n = 58)**
Energy (kcal)	2600	2152 ± 52^b^	2306 ± 49^b^	2282 ± 36^b^	2202 ± 28^b^	2300	1431 ± 54^a^
Protein (g)	85	118.2 ± 4.0^bc^	130.0 ± 3.2^b^	126.2 ± 2.7^b^	111.6 ± 2.0^c^	70	80.1 ± 3.5^a^
Fat (g)	57.8~86.7^3^	80.9 ± 2.7^b^	94.8 ± 2.9^c^	96.8 ± 2.3^c^	102.5 ± 2.0^c^	51.1~76.73	64.3 ± 2.5^a^
Carbohydrate (g)	357.5~422.5^4^	238.4 ± 8.9^b^	240.0 ± 8.2^b^	234.9 ± 5.8^b^	216.9 ± 3.8^b^	316.3~373.84	136.8 ± 6.8^a^
Dietary fiber (g)	30.2	8.2 ± 0.6^a^	10.4 ± 0.9^ab^	10.3 ± 0.5^bc^	14.0 ± 0.6^d^	30.2	7.8 ± 0.6^ac^
Vitamin A (μg RE^5^)	1200	1620 ± 173^b^	1304 ± 142^bc^	1146 ± 129^cd^	734 ± 60^ad^	700	255 ± 85^a^
Thiamine (mg)	1.8	1.5 ± 0.07^b^	1.5 ± 0.06^b^	1.6 ± 0.05^b^	1.7 ± 0.05^b^	1.3	1.2 ± 0.07^a^
Riboflavin (mg)	1.7	2.9 ± 0.17^bd^	3.2 ± 0.14^b^	2.7 ± 0.11^bc^	2.5 ± 0.11^cd^	1.2	1.3 ± 0.10^a^
Niacin (mg)	18	37.7 ± 1.84^b^	42.7 ± 1.48^b^	37.8 ± 1.10^b^	32.5 ± 0.75^c^	13	21.4 ± 0.85^a^
Vitamin C (mg)	130	59.1 ± 5.61^a^	68.7 ± 3.94^ab^	77.1 ± 4.09^b^	96.6 ± 4.44^c^	100	68.3 ± 3.98^ab^
Vitamin E (mg)	14	41.3 ± 1.36	45.0 ± 3.21	41.1 ± 1.12	40.4 ± 1.15	14	39.3 ± 0.84
Folate (μg DFE^6^)	500	441.6 ± 20.3^b^	455.2 ± 16.5^b^	470.0 ± 18.8^b^	441.1 ± 29.3^b^	400	286.8 ± 15.8^a^
Calcium (mg)	1200	428 ± 22^a^	454 ± 18^a^	595 ± 22^b^	544 ± 17^b^	800	404 ± 19^a^
Iron(mg)	25	34.7 ± 1.4^b^	37.7 ± 1.3^b^	34.8 ± 1.0^b^	30.0 ± 0.7^c^	20	21.6 ± 0.9^a^
Zinc (mg)	21.5	18.99 ± 0.79^b^	20.8 ± 0.77^b^	20.4 ± 0.59^b^	21.3 ± 0.59^b^	11.5	14.49 ± 0.68^a^
Selenium (μg)	65	145.3 ± 9.4^bc^	163.0 ± 7.3^c^	131.4 ± 5.8^b^	97.8 ± 4.6^a^	50	72.7 ± 5.8^a^
Iodine (μg)	200	162.2 ± 5.5^b^	220.7 ± 11.8^c^	214.6 ± 11.2^c^	248.3 ± 15.7^c^	150	200.3 ± 16.5^bc^
DHA (g)		0.24 ± 0.03^ab^	0.29 ± 0.03^b^	0.24 ± 0.02^ab^	0.17 ± 0.01^a^		0.13 ± 0.02^a^

^1^Values (mean ± SE) in the same row with different superscript letters (a, b, c, d) are significantly different, (*P <* 0.05 ~ 0.0001). Bonferroni adjustment for multiple comparisons of the lactating women compared with the control group of women (P < 0.01) and a repeated measures analysis of Greenhouse-Geisser adjustment for asphericity for 4 different time points comparison of the lactating women.

^2^RNI’s for control women are for a moderate level of physical activity.

^3^Conversion based on the Chinese RNI for fat providing 20%~30% of the total energy per day.

^4^Conversion based on the Chinese RNI for carbohydrate providing 55%~65% of the total energy per day.

^5^RE: retinol equivalent.

^6^DFE: dietary folic acid equivalent.

As shown in Tables 4, the mean intake of dietary carbohydrate in lactating women and the control group of women only consumed 61 ~ 67% and 43% of their RNI respectively. These values are based on the lowest recommended RNI of 357.5 g for lactating women and 316.3g for health adult control women with moderate physical activity. Based on the highest intake level of dietary carbohydrate for the Chinese RNI 422.5g for lactating women and 373.8 for the control group, both groups of women consumed only 51% ~ 57% and 37% of the recommended values throughout 3 months study, respectively. Carbohydrate consumption contributed 39% to 44% of the total energy in lactating women during first 3 months lactation and 38% in the control group (Table 4).

The mean fat intake of lactating women ranged from 29% to 44% higher than that of the lowest recommended levels of the RNI (57.8g/day) during the first 3 months of lactation, and ~10% greater than the highest level of the RNI (86.7g/day) during the last 3 time periods of lactation (Table 4). Fat consumption contributed ~34% ~ 42% of the total energy during the first 3 months of lactation. On lactation day 90, dietary fat, but not carbohydrate was the predominant source of energy. In the control group of women however, the main energy intake was fat (40%) and then carbohydrate (38%). The Chinese Dietary Guidelines [15,29] recommends the distribution of energy intake from three important nutrients as carbohydrate 55%~65%, fat 20%~30%, and protein 10 ~ 15%. Thus, the lactating and control group of women had a higher proportion of fat and lower proportion of carbohydrate intake than that recommended by the Chinese RNI [14,15].

As shown in Tables 4, lactating mothers consumed 31% to 53%, more protein than the RNI recommended level (85g/day) during the first 3 months of lactation. The highest protein intake was in lactating day 7 and 30 and lowest was in the lactating day 90. Overall difference between the 4 time-points of lactation was significant when all the data available were included in the analysis or for the 173 lactating women who had completed data sets were analyzed separately (P < 0.05, Table 4). The mean protein intake in the control group of women was 14% higher than the RNI (70g), however these levels were 25 ~ 38% lower than that of lactating mothers throughout the 3 months of lactation, whether adjusted or not for the BMI (P < 0.0001). The mean protein intake from animal food contributed 69 ~ 72% and 73% of total protein in the lactating and control group of women, respectively. Furthermore, there was greater than a 2 to 5-fold higher intake of poultry, eggs, milk and milk products in lactating mothers than in the control group of women (P < 0.0001, Table 3), whether adjusted for the BMI or not.

### Intake levels of selected vitamins and minerals

As shown in Table 4, the mean total dietary intake of vitamins and minerals from foods in the lactating women were lower than that recommended by the Chinese RNI ranging from 26% ~ 55% lower for vitamin C, 4% ~ 17% lower for thiamine, 6% ~ 12% lower for folic acid, 50% ~ 65% lower for calcium and 1% ~ 12% lower for zinc during the first 3 months of lactation. Of particular concern for the well being of the mother, and possibly the infant, was the low level of intake of vitamin C, folate and calcium. In contrast, there were elevated levels of intake above the RNI for riboflavin (48% ~ 87% higher), niacin (~2-fold higher), vitamin E (~3-fold higher), iron (20% ~ 50% higher) and selenium (1.5 ~ 2.5-fold higher). In lactating mothers, the mean intake of vitamin A, riboflavin, selenium, DHA and iron decreased during the course of lactation, while vitamin C and iodine increased. There was no significant change in the mean intake of vitamin B1, vitamin E, folic acid and zinc during the 3 months of lactation. In the control group of women, however, there was a low intake of vitamin A (36%), thiamine (92%), vitamin C (68%), folate (72%) and calcium (51%), compared to the recommended RNI. The intake of all vitamins and minerals were lower in the control group of women compared to the lactating mothers, in particular for thiamine, riboflavin, niacin, folic acid, iron and zinc during the first 3 months of lactation, whether adjusted for the BMI or not (P < 0.001). Also, the lactating and control group of women consumed only 27% ~ 46% and 26% of the amount of dietary fiber recommended by the RNI (30.2g/day), respectively.

## Discussion

The 24h dietary recall questionnaire is widely used as a tool for assessing food consumption, despite disagreements about its validity to accurately assess an individual’s mean intake (5–8). In this study, to circumvent this possible limitation, trained personnel visited each lactating mother in their homes on four occasions during the first three months of lactation, and recorded all food items they consumed. Our new findings showed that total energy and the mean intake of carbohydrate was 12-15% and 33 ~ 49% lower than the Chinese recommended RNI and protein and fat in lactating mothers ranged from 31 ~ 53% and 9-77% higher than the Chinese RNI respectively. Interestingly, the control group of women had similar nutrient intake patterns of lower energy (38%), lower carbohydrate (57% ~ 63%) and higher levels of protein (14%) than that recommended by the RNI for the moderate physical activity level of an adult. Thus, the main nutrient intake of both lactating mothers and the control group of women fails to meet Chinese RNI.

**Figure 1:**
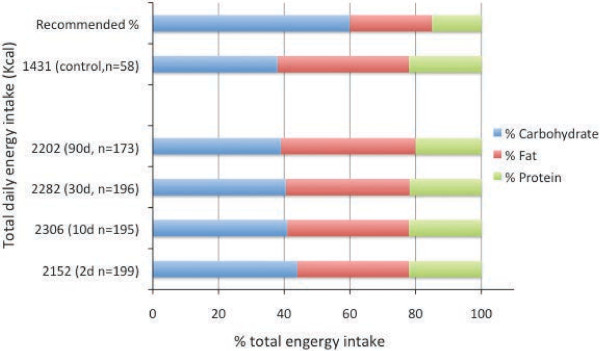
**Daily energy intake distribution from carbohydrate, fat, and protein**^**1**^**.**^1^The recommended distribution of daily energy intake in China is carbohydrates 55% ~ 65%, protein 12% ~ 16% and fat 20% ~ 30% respectively. The figure constructed using the media figure of the recommend levels of daily energy intake distribution from carbohydrates 60%, protein 15% and fat 25%.

The energy needs of a breastfeeding mother are increased because of milk production. Our findings showed that the mean daily energy intake of lactating mothers was 720 ~ 850 kcal higher than that of control woman during the first 90 days of lactation. Also the distribution of total calories from carbohydrate, 39% ~ 44%, protein, 20% ~ 23% and fat, 34% ~ 42% did not meet the recommended level of energy distribution (Figure 1). Interestingly the control group of women had similar energy distribution pattern as lactating women (Figure 1). Thus, the distribution of calories derived from the 3 major nutrients failed to meet a balanced diet as recommended by the Chinese RNI for both lactating and the control group of women (Figure 1).

Lactating women have an increased requirement for dietary protein intake for synthesis of the protein in breast milk, and for the growth, maintenance and repair of cells. As shown in Table 4, lactating mothers consumed 31% ~ 53% more protein than required by the Chinese RNI. Based on their body weight, this increased level of protein intake showed little change during the first 3 months of lactations, ranging from ~1.89g/kg to 2.2g/kg, which was higher than the 1.1g/kg reference daily intake (RDI) for lactating women in developed country e.g. Australia [30]. The comparative value for the control group of women was 1.51g/kg, which was higher than the 0.75g/kg recommended for 20 ~ 40 year-old non-pregnant women [30]. The American Heart Association does not recommend high protein diets because they may restrict healthy foods that provide essential nutrients and do not provide the variety of foods needed to adequately meet nutritional needs [31]. While these diets may not be harmful for most healthy adults for short periods of time there are, to our knowledge, no long-term scientific studies to support their overall efficacy and safety [31]. In particular, when high protein intake exceeds the body’s needs, this may place an individual at higher risk for kidney and liver disorders, osteoporosis and reduced vitamins, minerals, fiber and the uptake of other nutritional elements. All of these factors may thus lead to other potential health risks.

Although lactating women consumed ~70% of their protein from animal products, which is also a source of other essential nutrients, the animal foods usually contain a high content of saturated fatty acid (SFA’s). The consumption of a diet rich in SFA’s for a sustained period raises the risk of coronary heart disease (CHD), diabetes, stroke and several types of cancer [31]. The lactating group mothers consumed 9 ~ 77% more fat than the recommended level in China (Table 3,4). However, these lactating women consumed a diet higher in fish/shellfish and seaweed products (124 ~ 176g/day), which was higher than the recommended RNI (50 ~ 100g/day) [14]. Fish/shellfish are an important source of trace minerals, including iodine and selenium and long chain polyunsaturated fatty acid, e.g. DHA [32]. Fish are also a major source of vitamin D, which through neurotrophic, anti-inflammatory, and neuroprotective effects, may work synergistically with DHA to protect the brain [33]. DHA intake in lactating group women remained relatively constant during the first 3 month of lactation, ranging from ~290 mg/day at day 7 to ~170 mg/day at day 90 of lactation. This level is similar to that in the developed country e.g. ~160mg/day in Australia [30] and is higher than the general population in China, which is about 22.1mg/day in urban and 6.0mg/d rural area [34]. Recent studies have shown that the replacement of only 1% of energy from SFAs with polyunsaturated fatty acids (PUFAs) lowers cholesterol levels in the serum low-density lipoprotein (LDL), resulting in a reduction in the incidence of CHD by ≥2-3% [35]. Thus, the relatively high level of DHA intake in the lactating mothers may translate into a reduced risk of CHD, and it will be interesting to determine if long-term epidemiological studies validates this supposition.

It was unanticipated to find that lactating women during their first 3 months of lactation had 33% ~ 49% lower carbohydrate intake than that recommended by Chinese RNI (Table 4). The mean carbohydrate intake from cereals during the first 90 days of lactation was only 335g/d ~ 409g/d (Table 2). The variety of cereals included mainly rice and rice products that increased carbohydrate intake from 70.2 ~ 94.6g/d during the first 3 months of lactation. The mean intake of beans and tubers also increased during this period, while flour and flour products decreased (Table 2). The dietary habits and culture of inhabitants in southern Chinese usually consumed more rice and rice products; while in northern Chinese, the diet is richer in flour and flour products. Low carbohydrate diets have been used for weight control and the treatment of obesity [36]. It is therefore not a surprising finding that lactating mothers had little physical activity during their first 3 months of lactation, but their low carbohydrate diet, along with breastfeeding kept their BMI at <24 throughout the study. Surprisingly, the control group of women consumed only 37 ~ 43% of the recommended carbohydrate (RNI 316.3~373.8g/day), while their protein intake were ~12% higher than the RNI and their fat intake were in the normal range of the recommended levels (51.1-76.7 g/day, Table 4). Their total caloric intake (1431Kcal) was ~38% lower than the recommended daily RNI of 2300Kcal for women with a moderate level of physical activity (Table 4). Thus, the lower total caloric intake, resulting from the decreased carbohydrate, increased protein and the median level of fat consumption, served to keep the BMI of all control women in the normal range recommended [37]. A healthy diet enriched in fresh fruits, vegetables, cereals and grains, and low in fat, coupled with regular physical activity can help many people manage and maintain adequate health and well being and a decreased health risk from CHD. Based on our findings, it is evident that further long-term studies are required to follow up on the health and disease risks for the population of Chinese mothers and infants who do not receive the RNI recommended level of nutrients required to sustain health.

Lactating mothers should have a slightly increased requirement for most nutrients compared to women who are not pregnant or lactating, as many vitamins and minerals in a breastfeeding mother's diet are transferred into the breast milk [35]. We found in the lactating mothers however, that the intake of several important vitamins and minerals during the first 90 days of lactation were 6% ~ 75% lower than the RNI, including thiamine, vitamin C, folate, calcium and zinc, as summarized in Tables 4. Thiamine and folate are important B vitamins that are involved in many cellular processes critical for healthy growth and development [38]. Vitamin C is an essential nutrient, and plays an important role as an antioxidant to protect the body against oxidative stress [39]. Similarly, zinc is a co-factor for many enzymes and also functions to help maintain structural integrity of proteins and to regulate gene expression [40]. Long-term deficiencies in these nutrients can have a significant deleterious impact on health, causing deficiency diseases, health-threatening conditions and some common chronic systemic diseases [41].

Similarly, we also discovered that the intake of dietary fiber in the lactating mothers was less than 1/2 ~1/4 of the level recommended by the RNI during throughout study. Conversely, the intake levels of riboflavin, niacin, vitamin E, iron and selenium were 50% to 3-fold higher than the RNI during the first 3 months of lactation, as summarized in Table 4. In humans, there is no evidence for riboflavin toxicity produced by excessive intakes, as any excess at nutritionally relevant doses is excreted in the urine [42]. Vitamin E, a fat soluble vitamin that is stored in lipophilic tissues and is also an antioxidant, appears to be relatively non-toxic in healthy adults. However, its long-term safety is questionable following results showing a possible increase in mortality and in the incidence of heart failure [43]. The high intake of niacin, iron and selenium in our subjects were likely caused by the high protein intake from animal food. Thus, the self-selected diet of lactating mothers in southeast coastal China is unbalanced and fails to meet the recommended Chinese RNI for lactating mothers. On this basis, we postulate that the long term unbalanced dietary intake may increase the potential health risks for both lactating mothers and their babies. Accordingly, we recommend that dietary fiber, thiamine, vitamin C, folate, calcium and zinc levels should be increased in the diet of Chinese lactating women and that the total levels of niacin and selenium should be decreased by reducing fatty animal food intake. This important observation should be of concern to health care professionals charged with overseeing the health and well being of mothers and children in China. To our knowledge, this is the first systematic study to monitor and evaluate the total and complete level of food intake in lactating mothers during their first 90 days of lactation in the Southeast coastal region of China.

## Conclusions

In many cases, the diet of the lactating and control group of women does not meet the Chinese RNI and, as a consequence, may have a negative impact on the quality of maternal milk and affect the potential health of mothers and infants. Nutritional education is essential for both lactating and non-lactating women. The population of China is over 1.34 billion people with 56 distinct ethnic groups. Each ethic group have their own dietary habits and cultural differences, such as northern China people prefer to eat more noodles or steam buns and hot spicy foods including chilies, onions, and garlic, while the southern Chinese people in general consume large amounts of rice and mild and cooling foods. The RNI needs to more adequately reflect the regional dietary habits and culture. A single national RNI is not applicable to all regions of China. Increased intake of fresh vegetables, fruits multivitamin and calcium supplements should be recommended by Chinese health officials to all women, and to those who do not consume adequate amounts of these foods and supplements. A healthy, balanced diet for lactation is important for both mother and infant in order to have a positive, long-term effect on the outcome of their health and resistance to disease. The more cross-country surveys are needed in future.

## Competing interests

None of the authors have any competing interest with respect to the study.

## Authors’ contributions

BW, conceptualized and designed the study; BW and FAT contributed to the analyses and interpretation of the results and final writing of the manuscript; HJC, PW, and JM collected the data, HJC and PW contributed to the first draft the manuscript, YFH and HJC carried out the statistical analyses. All authors read and approved the final manuscript.

## Pre-publication history

The pre-publication history for this paper can be accessed here:

http://www.biomedcentral.com/1472-6874/12/18/prepub
